# Cytomegalovirus Retinitis: Clinical Manifestations, Diagnosis and Treatment

**DOI:** 10.3390/v16091427

**Published:** 2024-09-07

**Authors:** Jing Zhang, Koju Kamoi, Yuan Zong, Mingming Yang, Yaru Zou, Miki Miyagaki, Kyoko Ohno-Matsui

**Affiliations:** Department of Ophthalmology and Visual Science, Graduate School of Medical and Dental Sciences, Tokyo Medical and Dental University, Tokyo 113-8510, Japan; zhangj.c@foxmail.com (J.Z.); zongyuan666@gmail.com (Y.Z.); yangmm-12@outlook.com (M.Y.); alicezouyaru519@gmail.com (Y.Z.); miyagakimk@gmail.com (M.M.); k.ohno.oph@tmd.ac.jp (K.O.-M.)

**Keywords:** cytomegalovirus, retinitis, antiviral treatment, differential diagnosis, immunodeficiency

## Abstract

Cytomegalovirus (CMV) retinitis is the most common eye disease associated with CMV infection in immunocompromised individuals. The CMVR may initially be asymptomatic; however, relatively mild vitreous inflammation at the onset may be an important differential point from other diseases in HIV patients. Fundus photography, CD4 T-cell count, and telemedicine could be used to screen and monitor the high-risk population, particularly in resource-limited regions. Retinitis generally starts in the peripheral retina and advances toward the posterior pole, which could develop to the characteristic “pizza pie” appearance marked by central retinal necrosis and intraretinal hemorrhage. CMVR causes vision loss if left untreated, and early antiviral therapy significantly reduces the risk of vision loss. Alongside traditional antiviral treatments, immunotherapies including CMV-specific adoptive T-cell therapy and CMV immunoglobulin (CMVIG) are emerging as promising treatment options due to their favorable tolerability and reduced mortality. This review comprehensively examines CMV retinitis, encompassing the clinical features, differential diagnosis, laboratory tests, and updated treatment strategies to inform clinical management.

## 1. Introduction

Cytomegalovirus (CMV) is a ubiquitous herpesvirus transmitted via blood, body fluids, organ transplants, and vertical transmission. Over 40% of the general population has been infected [1], although initial infection is often asymptomatic, with the virus remaining latent [2]. The eye is a known latent site for CMV [3], and common ocular manifestations in the general population include anterior uveitis, corneal endotheliitis, and elevated intraocular pressure [4]. 

In immunocompromised individuals, CMV can cause severe opportunistic infections, with retinitis being the most common ocular manifestation [5], affecting approximately 20–40% of human immunodeficiency virus (HIV)-positive patients [6]. Beyond HIV, human T-cell leukemia virus type 1 (HTLV-1) is another retrovirus linked to CMV retinitis (CMVR). With an estimated 20 million global infections, HTLV-1 is prevalent in Japan and among Australian Aboriginal adults [7]. Discovered in the 1980s, HTLV-1 is associated with adult T-cell leukemia/lymphoma (ATL) and various ocular diseases [8,9,10,11,12,13,14,15]. According to a nationwide survey [16], CMVR is the most frequent infectious manifestation among ATL patients, occurring more often than in other leukemias [17].

CMVR has a global distribution but exhibits varying incidence rates, with lower prevalence in HIV-infected Latin American and Africa and higher rates in Asia [18]. The low prevalence in Latin America may relate to free and universal access to ART [19]. The low prevalence in Africa suggests HIV patients either dying from other diseases before reaching severe immunosuppression or having very brief survival at this stage [20]. The high prevalence in Asia might be linked to genetic factors [21]. The initial CMVR symptoms are nonspecific, often including blurred vision, floaters, and flashing lights [22]. Lesions typically begin peripherally and progress toward the posterior pole [23]. When left untreated, CMVR can lead to irreversible vision loss through retinal detachment and functional damage [24]. CMVR diagnosis relies on fundus examination and diagnostic tests [25]. Early symptoms being atypical, risk factors like CD4+ T-cell counts below 50 cells/µL in HIV patients should prompt early diagnosis [26], and CD4+ T-cell can also be used as one of the indicators for CMVR screening in developing countries [27].

Similar to HIV, HTLV-1 affects CD4+ T-lymphocytes, increasing susceptibility to opportunistic infections like CMVR [12,28]. T-cell monitoring is crucial in these populations. Antiviral therapy remains the cornerstone of CMVR treatment, controlling viral replication, reducing inflammation, and slowing disease progression [29]. Combination ART has significantly reduced CMVR incidence and morbidity in HIV patients [30]. Notably, studies have suggested that patients with preexisting CMV retinitis who are treated with HAART may develop immune recovery uveitis (IRU) [31], which is the most common form of immune reconstitution inflammatory syndrome and a common cause of new vision loss [32]. It is likely the result of previously exposed antigens from CMVR being recognized by the enhanced immune response [33].

The rising number of non-HIV immunocompromised individuals [34], including those with autoimmune diseases and organ transplants, represents a growing CMVR risk population. A study published in 2021 showed that 11.3% of patients with CMV viremia developed CMV retinitis after transplantation, with a significantly higher mortality rate relative to the hematopoietic stem cell transplantation (HSCT) group compared with the (solid organ transplantation) SOT group, implying that CMVR should be brought to wider attention [35]. While immunotherapy research advances, potential adverse effects and resistance in immunocompromised patients remain challenges [36,37,38]. Early diagnosis and treatment are essential for optimal visual outcomes [39], as delayed intervention hinders disease control and vision preservation. This review aims to provide a theoretical foundation for early diagnosis and appropriate CMVR management through a systematic examination of clinical manifestations, diagnostic methods, and treatment options.

## 2. Clinical Features of CMVR Optimal Outcomes

The CMVR may initially be asymptomatic, and relatively mild vitreous inflammation at the onset may be an important differential point from other disease in HIV patients [40]. As the disease progresses, patients may experience floaters, flashes of light, and blind spots [41]. Retinopathy typically begins in the peripheral retina and gradually advances toward the posterior pole at an average rate of 24 mm per day [42]. When untreated, the disease can lead to retinal detachment, a major cause of vision loss [29]. 

**Figure 1:**
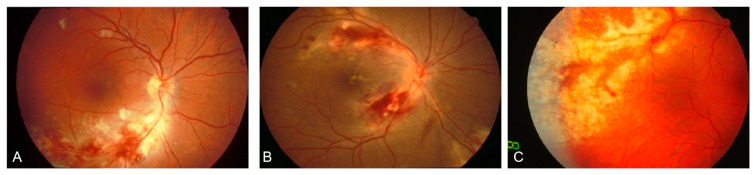
Fundus photograph of cytomegalovirus retinitis: (**A**) wedge-shaped appearance with the apex “pointing” toward the optic disc. (**B**) a “hemorrhagic” appearance involving the posterior pole, characterized by retinal necrosis and edema, intraretinal hemorrhage and “satellite lesions” at the border. (**C**) cytomegalovirus retinitis involving the periphery, characterized by a “granular” appearance and without hemorrhage. (Modified from Standardization of Uveitis Nomenclature (SUN) Working Group, American journal of ophthalmology, 2021, 228: 245–254 [43], under a Creative Commons licence CC BY).

According to the recent SUN criteria, the characteristic CMV retinitis include wedge-shaped area of retinitis (Figure 1A), hemorrhagic retinitis (Figure 1B) and granular retinitis (Figure 1C) with no to mild vitritis [43]. The hemorrhagic type presented with a more extensive area of retinal edema and necrosis, commonly found in the posterior pole. While the granular type had a “granular” appearance, which is more prevalent in the peripheral retina [43]. When central necrosis accompanies intraretinal hemorrhage, the “pizza pie” or “ketchup–cheese retinopathy” appearance may occur [44]. 

**Figure 2:**
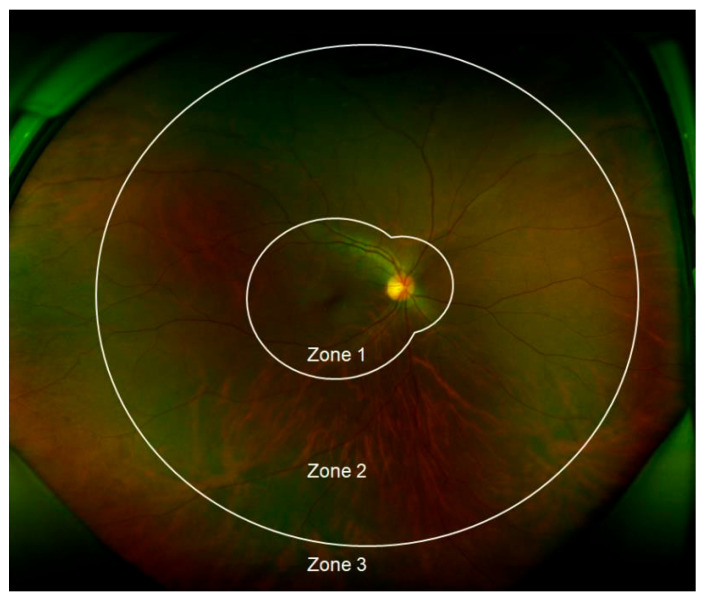
Cytomegalovirus (CMV) retinitis zones: Zone 1 encompasses the area within 1500 µm of the optic nerve or 3000 µm of the fovea. Zone 2 extends from the outer boundary of Zone 1 to the equator, as determined by the vortex veins. Zone 3 covers the peripheral retina from the equator to the ora serrata (Photo by Kwon H J, et al., Microorganisms, 2021 [48], under a Creative Commons licence CC BY 4.0).

To categorize retinal involvement, CMVR is divided into zones (Figure 2). Zone 1 encompasses the area within 1500 μm of the optic nerve head and 3000 μm from the fovea. Zone 2 extends from Zone 1 to the equator, and Zone 3 includes the area beyond Zone 2, reaching the ora serrata [45]. Involvement of the macula or optic nerve (Zone 1) results in significant visual impairment [46]. Compared to other regions, lesions in Zone 1 are more challenging to visualize, often requiring more injections and associated with higher complication rates [47].

CMVR is prevalent in immunocompromised individuals, including HIV patients and organ transplant recipients. Epidemiological comparisons between these groups reveal distinct characteristics. HIV patients exhibit a higher male predominance and younger age compared to non-HIV patients [49,50], potentially influenced by the age distribution of non-HIV patients [51]. Notably, decreased lymphocyte counts in HIV patients serve as an early CMVR indicator [50,52]. 

Non-HIV patients often experience ocular inflammation, including vitritis, retinal arteritis, and vascular occlusions, with the severity correlating to the immune suppression levels [53,54,55]. Treatment approaches differ, with HIV patients requiring fewer intravitreal injections compared to non-HIV patients [49,50]. Recurrence rates are lower in HIV patients [49,50], who also exhibit longer survival times [49,50]. While some studies report similar CMVR clinical symptoms between the groups [49,56], others suggest a higher incidence of central retinitis in non-HIV patients with more bilateral and central lesion involvement. Despite this, non-HIV patients tend to have better treatment outcomes and lower complication rates [57]. The differences in these study conclusions may be primarily influenced by patients’ immune levels and the treatment regimens they receive. Therefore, future research and clinical practice should place a greater emphasis on managing and treating CMVR across different patient populations, particularly by developing personalized treatment plans based on patients’ specific immune status and therapeutic needs. 

## 3. Differential Diagnosis of CMVR

CMVR’s atypical fundus manifestations often lead to misdiagnosis. Table 1 summarizes other retinitis-prone diseases, including herpesvirus infections, syphilis, tuberculosis, and toxoplasmosis. 

In HIV patients, progressive outer retinal necrosis (PORN) and acute retinal necrosis (ARN) are common vision-threatening retinal diseases alongside CMVR [58]. PORN involves multiple deep, sharply demarcated white retinal lesions with optic nerve involvement and minimal vitreous infiltration [59]. ARN presents with yellow-to-white retinal lesions and marked vitreous and anterior chamber infiltration [41]. 

Syphilitic retinitis, often misdiagnosed as viral retinitis, is confirmed through systemic investigations [60]. A characteristic diaphanous or ground-glass retinitis with creamy yellow superficial retinal precipitates suggests syphilis [61]. 

Mycobacterium tuberculosis is the most common opportunistic infection associated with AIDS, and although ocular tuberculosis is uncommon, it still warrants a differential diagnosis in developing countries [62]. Tuberculous retinitis is common in TB-associated intraocular inflammation [63], which involves vitreous opacification, gray-white retinal lesions, and focal retinal vasculitis [64,65]. Due to the nonspecific symptoms and potentially low pathogen load, diagnosing tuberculosis may require tuberculin skin tests or chest radiographs in addition to polymerase chain reaction (PCR) [66]. 

Toxoplasmosis presents with variable gray-to-white or pale-yellow lesions [67]. Active lesions exhibit foci of retinochoroiditis with poorly defined borders, often near scars [68]. Periphlebitis is common, and active lesions typically result in atrophic scars with pigmentation [69]. 

In addition to the diseases mentioned, other conditions may necessitate differential diagnosis. One case report described a CMVR patient diagnosed with bilateral intraocular lymphoma, with a history of diffuse large B-cell lymphoma (DLBCL) and atypical fundus findings [70]. The patient exhibited yellow-white deep retinal lesions, sparse superficial retinal hemorrhages, and perivascular exudates. Furthermore, other case reports have documented the occurrence of CMVR during chemotherapy for DLBCL [71,72] CMVR diagnosis. Consequently, a thorough evaluation that includes assessing necrotizing retinitis with poorly defined borders, immune compromise, characteristic clinical fundus findings, or evidence of intraocular CMV infection, while excluding other differential diagnoses, can facilitate a prompt and accurate diagnosis of CMVR [43].

## 4. Laboratory Test and Screening of CMVR

Early CMVR symptoms are often nonspecific, necessitating a combined approach of medical history and related examinations for diagnosis. A machine learning study demonstrated the effectiveness of laboratory tests, retinopathy, immune compromise history, and infection exclusion (e.g., syphilis and herpesvirus) in diagnosing and reducing misclassification [43]. Early laboratory testing in at-risk individuals, combined with ophthalmologic evaluation for retinal edema, macular lesions, and hemorrhagic exudates, is crucial.

The aqueous humor PCR assay is commonly used for detecting active CMV viral replication and aiding CMVR diagnosis [73]. A comprehensive PCR assay examining ocular samples, including viral genomic DNA, and multiplex PCR analysis of viruses and *Toxoplasma gondii* in aqueous humor, has shown clinical effectiveness in endophthalmitis cases [74]. Additionally, broad-spectrum real-time fluorescence quantitative PCR for aqueous humor DNA (rDNA) is a reliable tool for diagnosing ocular infections and screening for intraocular infections. While blood or urine tests can detect CMV viral infection, negative results do not exclude CMV, necessitating a combined interpretation. 

**Figure 3:**
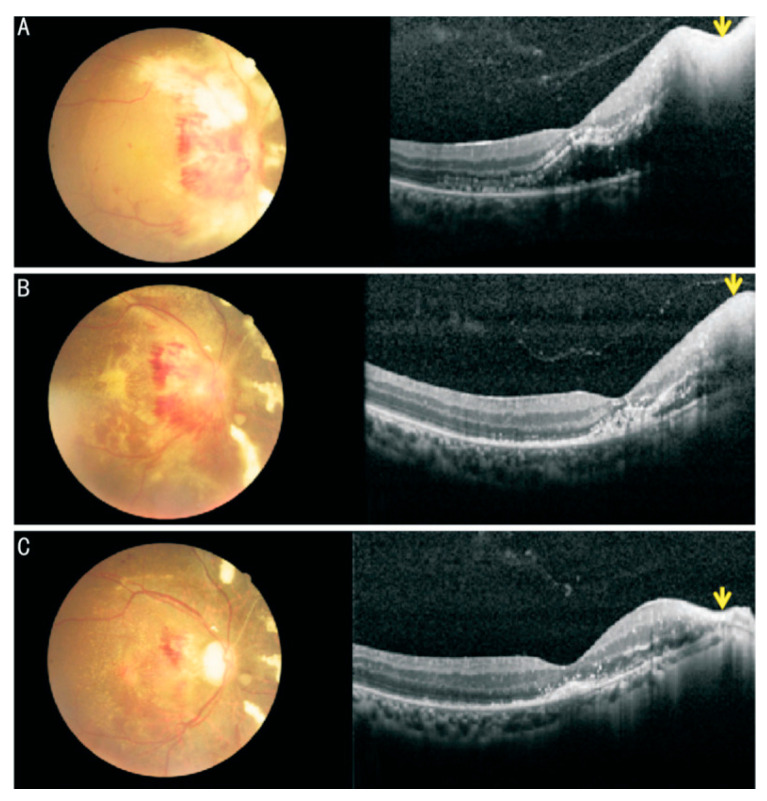
Fundus photographs and OCT images of CMVR and cytomegalovirus papillitis: (**A**) First visit: fundus photography showed yellowish white retinal necrosis and retinal hemorrhage around the disc; OCT showed exudative retinal detachment in macular area and significant thickening and hyperreflective in temporal retina of optic disc with full-thickness disruption of retinal architecture (yellow arrow). (**B**) Two weeks: after 2 wk of anti-cytomegalovirus therapy, retinal necrotic lesion has disappeared on fundus photograph, OCT showed subretinal fluid absorption and the edema of the necrotic lesion relief (yellow arrow). (**C**) Six weeks: fundus photography showed optic atrophy and the retinal necrosis and most of the retinal hemorrhage were absorbed; OCT showed complete absorption of subretinal fluid and retinal thinning in temporal retina of optic disc (yellow arrow). (Photo by Sheng, Yan, et al. International Journal of Ophthalmology, 2020, 13(11): 1800 [77], under a Creative Commons licence CC BY 4.0).

Early CMVR lesions may mimic HIV retinopathy on fundus photography but appear distinct on optical coherence tomography (OCT) images [75]. CMVR’s necrotic and thinned retinal layers create a lace-like OCT appearance [72], contrasting with HIV retinopathy’s inner retinal lesions and the preserved outer layers on OCT imaging [76]. CMVR OCT in AIDS patients was categorized into typical and atypical presentations. In the active phase, the typical presentation was characterized by significant thickening of the retina with hyperreflective lesions and destruction of all layers of the retinal structure with vascular enlargement (Figure 3A), while the atypical type showed the destruction of all layers of the retina as well, but no thickening or slight thinning. The choroid, vitreous, and retinal vessels were not significantly involved. While in the healing stage, the retina is thinner and both types of retinal layers are disrupted, as shown in Figure 3C [77]. Spectral domain OCT effectively diagnoses, manages, and predicts CMVR outcomes, and can be used as an effective test for CMVR management [78]. 

In resource-limited settings, fundus photography and telemedicine offer the potential for CMVR screening and monitoring in high-risk populations. Machine learning models have demonstrated accuracy in diagnosing CMVR from fundus photographs [43], with sensitivity and specificity reaching 88.2% and 100% in Zone 1 retinitis, respectively [79]. These findings highlight the potential of fundus photography and telemedicine for preventing CMVR blindness.

## 5. Treatments and Updates of CMVR

Prompt diagnosis of CMVR through immune history, clinical symptoms, and PCR analysis is crucial for initiating timely treatment to reduce retinal detachment and maintain quality of life. Table 2 summarizes recent therapeutic advancements in CMVR to guide treatment selection based on lesion severity and patient health.

Antiviral treatments have significantly reduced CMVR incidence and severity, with effective anti-CMV therapy lowering vision loss risk [80] and improving survival [81]. Initial treatment typically involves first-line drugs like ganciclovir (GCV), valganciclovir (VGCV), cidofovir (CDV), and foscarnet (FOS). GCV, VGCV, CDV, and FOS inhibit CMV DNA polymerase UL54, slowing down DNA synthesis [82]. GCV, VGCV, and CDV indirectly inhibit polymerase by incorporating into viral DNA [83] or acting as nucleotide analogs [84]. FOS uniquely inhibits viral DNA polymerase by binding to its pyrophosphate binding site [85]. Letermovir (LET), a CMV-terminase inhibitor preventing viral DNA packaging [86], is used for drug-resistant CMVR in patients with acute immunodeficiency syndrome [87]. Maribavir (MBV), an oral CMV DNA UL97 kinase inhibitor, offers a novel therapeutic option [88]. Administration routes include systemic (intravenous and oral) and topical (intravitreal and intraocular sustained-release implant) methods. Beyond monotherapy, combination therapies like GCV + FOS have gained attention in recent years [89,90,91]. 

GCV is the first-line treatment for CMV, offering various administration routes including oral, intravenous, vitreous, and intraocular implants [92]. The recommended oral GCV regimen for preventing CMV disease in allograft recipients with normal renal function is 1000 mg three times daily. Intravenous GCV should be administered continuously over 1 h at a dose of 5 mg/kg every 12 h for 7 to 14 days [93]. This should be followed by 5 mg/kg once daily, 7 days per week, or 6 mg/kg once daily, five times per week. Extensive clinical data support GCV’s efficacy in preventing CMV infections and recurrences [94]. For patients in developing countries, repeated intravitreal injections of GCV, although time-consuming and labor intensive, have proven to be very effective, relatively safe, and extremely affordable [95]. However, systemic GCV may induce myelosuppression, neutropenia, anemia, thrombocytopenia, and liver toxicity [96], while topical administration can lead to vitreous hemorrhage and retinal detachment, necessitating close monitoring [97]. For zone 1 retinitis, intravitreal drug injections or intraocular implants of slow-release GCV reservoirs are considered [81], with intravitreal injections potentially being more cost-effective [81]. 

VGCV, an oral prodrug converted to GCV, offers convenient dosing and improved patient compliance compared to intravenous GCV [98]. Research has shown that a twice-daily dose of 900 mg of oral VGCV for induction therapy in CMVR patients has an efficacy and safety profile comparable to that of intravenous ganciclovir [99]. Nevertheless, VGCV carries risks of neutropenia, anemia, and thrombocytopenia [100]. Despite these adverse effects, it remains a valuable option under careful medical supervision. 

CDV, administered intravenously and intravitreally, achieves high drug concentrations for effective CMV targeting [92,101,102]. It is suitable for treating patients with GCV resistance [103], and its lower cost [104,105] makes CDV advantageous for managing resistant CMV infections. The recommended dosing regimen for CDV in the treatment of CMVR includes an induction phase of 5 mg/kg administered intravenously over 1 h, once a week for 2 weeks, followed by a maintenance phase of 5 mg/kg administered intravenously every 2 weeks [106]. Furthermore, a 20 µg dose of cidofovir intravitreal was effective in preventing the progression of CMV retinitis [107]. However, CDV can cause proteinuria, renal failure, neutropenia after systemic treatment [108], while ocular hypotony, vitreous hemorrhage, retinal detachment, and intraocular inflammation may occur after intravitreal CDV [109]. Thus, careful monitoring is essential while using CDV. 

FOS, with a different mechanism of action than GCV, serves as an alternative treatment option for intravenous and intravitreal administration [110], particularly for drug-resistant patients [90]. The recommended dose of foscarnet is 60 mg/kg IV over 2 h every 12 h for 14 days as induction therapy. If CMV antigenemia remains detectable, a maintenance dose of 90 mg/kg/day IV over 3 h is recommended until antigenemia clears [111]. A retrospective study found that intravitreal injections of FOS (2.4 mg in 0.1 mL per injection) in immunocompromised patients with CMV retinitis, administered twice weekly during induction therapy and once weekly during maintenance therapy, resulted in stable vision in 61% of patients and improved vision in 5%. This suggests that it may be an effective alternative treatment [112]. Although effective, FOS can impair renal function, induce anemia, and cause electrolyte disturbances [113], necessitating the close monitoring of renal function and electrolytes. 

In addition to traditional antivirals, LET and MBV have emerged as promising treatment options. LET targets the CMV terminase complex, offering a novel therapeutic approach. Compared to traditional antivirals, oral or intravenous LET is generally well tolerated [114], making it a suitable choice for preventing CMV infections and diseases in transplant recipients [115]. The recommended dose of LET for CMV retinitis is 480 mg once daily, continued for up to 100 days post-transplant. It can be administered either orally or through an IV infusion over 1 h [116]. However, LET is currently approved for prophylaxis, and its efficacy diminishes with increasing viral load, limiting its therapeutic range [88]. Nonetheless, LET represents a significant advancement in preventing CMV in hematopoietic stem cell transplantation recipients due to its effective prophylaxis and improved safety profile. 

MBV, an oral drug primarily used for CMV in HSCT or SOT recipients [117], has gained recent attention. Its unique mechanism of action renders it effective against traditional antiviral-resistant CMV strains. The recommended dose of MBV is at least 400 mg twice daily, demonstrating similar efficacy to VGCV in clearing CMV viremia among HSCT or SOT [118]. While generally well tolerated orally, MBV exhibits poor retinal penetration, with a vitreous human-to-plasma ratio of up to 0.28 [119]. Despite this limitation, MBV’s resistance profile and oral administration make it a valuable option for treating refractory or drug-resistant CMV infections in transplant recipients. The clinical use of LET and MBV mitigates the systemic toxicity and resistance associated with traditional antivirals, offering new possibilities for combination therapy.

In contrast to traditional antiviral therapies with potential serious side effects, immunotherapy, particularly CMV-specific adoptive T-cell therapy, is gaining traction. Studies indicate that infusing CMV-specific T cells can restore protective immunity [120]. For hematopoietic stem cell transplant recipients, immune reconstitution using these T cells effectively reduces viral reactivation-related morbidity and mortality [121]. Clinical trials have demonstrated the therapeutic potential of autologous T cells for treating recurrent or antiretroviral-resistant CMV infection in SOT recipients [122]. Compared to antiviral therapies requiring laboratory monitoring for adverse effects and drug resistance, this approach rebuilds immunity with fewer side effects [123]. A report shows that CMV-specific T-cell therapy can effectively and durably alleviate both virologic and clinical symptoms in a 21-month-old male with Wiskott–Aldrich syndrome and CMVR [124]. While donor selection complexity, time, and cost constraints currently limit widespread adoption, CMV-specific adoptive T-cell therapy holds promise as a safe and effective CMVR treatment.

CMVIG offers another immunotherapy approach, providing passive immunity through intravenous administration to control CMV infections, especially in organ transplant recipients [125,126,127,128]. A meta-analysis demonstrated CMVIG’s effectiveness in preventing CMV disease and reducing CMV-related deaths, with a nearly threefold decrease in incidence among SOT recipients [129]. Derived from plasma with high CMV antibody levels, CMVIG neutralizes circulating CMV particles, promoting viral elimination before host cell infection [130]. Additionally, high-titer CMVIG preparations possess immunomodulatory properties enhancing antiviral drug efficacy and potentially inhibiting indirect CMV infection effects, complementing the antiviral therapies [130,131]. Combination therapy with CMVIG and GCV significantly enhance visual outcomes and reduce intraocular CMV viral load in cases of vision-threatening CMVR [132]. While CMVIG exhibits minimal adverse effects [133], limitations include infusion reactions, donor selection challenges, and cost [134]. Despite these drawbacks, CMV immunoglobulin remains a valuable tool for reducing CMV infection risk and complications in susceptible populations.

In addition to antiviral therapy and immunotherapy, vitrectomy is the standard treatment of retinal detachment secondary to CMVR with 56–94% silicone oil repair effectiveness in HIV patients [135]. A 2021 study found significant improvements in best-corrected visual acuity (BCVA) at 1, 3, and 6 months after vitrectomy in AID patients [136]. While the retinal attachment rate was 87.2% at 1 month and 82.1% at 3 months postoperatively, it decreased to 71.8% at 6 months. This suggests that the retinal attachment rate decreased with the duration after surgery, and that patients with CD4+ cell counts below 50 cells/μL had a poorer prognosis in terms of reattachment rate. Another study also noted that vitrectomy offers a viable opportunity to maintain or improve vision and quality of life for CMVR retinal detachment in AID patients [137]. 

Of note, it is essential to monitor key parameters to ensure effective management and minimize complications during the CMV treatments. Studies have shown that the final CD4+ T-cell count > 50 cells/mm^3^ is associated with improved survival in AIDS patients, highlighting the importance of regular monitoring to assess treatment effectiveness [6]. Serial renal function tests are crucial due to the potential nephrotoxicity of antiviral therapies like GCV or FOS [5]. Additionally, complete blood counts (CBC) should be monitored to detect bone marrow suppression, a possible side effect of these antiviral treatments [138]. Previous studies have identified the positive correlation between the extent of the retinal area affected by CMV retinitis and the aqueous CMV load [139]. Thus, viral load testing can help assess treatment effectiveness and detect resistance. Liver function tests should also be conducted periodically for monitoring hepatotoxicity [140]. Finally, regular ophthalmologic examinations are essential to evaluate the progression of retinitis and the response to treatment [141].

## 6. Conclusions

CMVR is a significant ocular complication in immunocompromised individuals, often presenting with nonspecific symptoms like floaters, flashes of light, and blurred vision. When left untreated, it progresses to retinal detachment and vision loss, emphasizing the urgency of early detection and intervention. A comprehensive evaluation that includes assessing necrotizing retinitis with poorly defined borders, immune compromise, characteristic clinical fundus findings, or evidence of intraocular CMV infection, while excluding other differential diagnoses including PORN, ARN, and syphilitic retinitis, can facilitate a prompt and accurate diagnosis of CMVR. Fundus photography, CD4+ T-cell count, and telemedicine offer promising tools for early screening and monitoring, especially in resource-limited countries. The first line of clinical treatment for CMV infection is antiviral therapy, primarily with GCV. However, due to its excessive side effects, it is essential to monitor CD4+ T-cell count, renal function, complete blood counts, viral load, liver function, and ophthalmologic examinations to ensure effective management and minimize complications. Alternative treatments like LET, MBV, and T-cell immunotherapy have gained increasing awareness for good tolerability and may represent the future direction of research in CMV treatment. Given the substantial risk of blindness in immunodeficient patients, early diagnosis and continuous monitoring warrant greater attention.

## 7. Future Directions

While traditional antiviral therapies form the mainstay of treatment, their potential for serious side effects necessitates careful monitoring. Recent advancements in combination therapies and immunotherapies, such as CMV-specific T cells and CMVIG, provide alternative treatment options. The latent nature of CMV infection in the eye underscores the importance of vigilant screening and prompt management to prevent vision-threatening CMVR.

## Figures and Tables

**Table 1:** Differential diagnosis of CMV retinitis.

Infection	Lesion Color	Lesion Morphology	Other Manifestations
CMV ^1^ Retinitis	Yellow to white retinal lesions, usually with hemorrhage	Superficial, granular, with central pigmentary	CD4+ count < 50 cells/mL in HIV patients
PORN ^2^ (HSV ^3^-1/-2, VZV ^4^)	White retinal lesions	Multiple deep and sharply demarcated	Optic nerve involvement and minimal vitreous infiltration
ARN ^5^ (HSV-1/-2, VZV)	Yellow to white retinal lesions	Massive whitish edema fibrotic bands	Marked vitreous and anterior chamber infiltration
Syphilitic Retinitis	Yellow to orange retinal lesions	Creamy superficial retinal precipitates Placoid lesions and autofluorescence	Argyll Robertson pupil
Tuberculous Retinitis	Gray-white retinal lesions	Focal retinal vasculitis	Vein occlusion Active retinal periphlebitis
Toxoplasmosis	Variable gray to white or pale-yellow lesions	Retinochoroiditis pigmented scar Adjacent vitritis	Peri-phlebitis

Abbreviations: ^1^ CMV, Cytomegalovirus; ^2^ PORN, progressive outer retinal necrosis; ^3^ HSV, herpes simplex viruses; ^4^ VZV, Varicella Zoster virus; ^5^ ARN, acute retinal necrosis.

**Table 2:** Treatments for CMV retinitis.

Medication	Mechanism	Administration	Advantages	Limitations
Ganciclovir (GCV)	CMV DNA polymerase UL54 Inhibitor	Intravenous Oral Intravitreal Intraocular implant	Multiple routes of administration Effectively prevents CMV infection and recurrence	Strong pill burden including myelosuppressant and liver toxicity
Valganciclovir (VGCV)	CMV DNA polymerase UL54 Inhibitor	Oral	Low pill burden Convenient dosing	Neutropenia, anemia and thrombocytopenia
Cidofovir (CDV)	CMV DNA polymerase UL54 Inhibitor	Intravenous Intravitreal	Least expensive suppresses exacerbations	Proteinuria renal failure, neutropenia and uveitis Ocular hypotony
Foscarnet (FOS)	CMV DNA polymerase UL54 Inhibitor (directly)	Intravenous Intravitreal	Combination therapy with Ganciclovir An alternative to Ganciclovir Less bone marrow suppression	Impaired renal function, anemia, and electrolyte disturbances Vitreous hemorrhage retinal detachment
Letermovir (LET)	CMV-terminase inhibitor	Oral Intravenous	Well tolerated Combination therapy with Ganciclovir	Approved only for prophylaxis Reduced efficacy with high viral loads
Maribavir (MBV)	CMV kinase UL97 inhibitor	Oral	No significant renal, hematologic, or hepatic toxicity	Poor penetration to retina Resistances
CMV-specific adoptive T-cell therapy	Restore CMV-specific T-cell immunity	Intravenous	Restore immunity Well tolerated Alternative option in drug resistant	Complex donor selection Costly
CMV immunoglobulin (CMVIG)	Restore passive CMV-specific T-cell immunity	Intravenous	Restore immunity Well tolerated Combination therapy	Complex donor selection Costly Infusion reactions

Abbreviations: CMV, Cytomegalovirus.

## Data Availability

All data related to this study are presented and published here.
